# Knowledge, attitudes, and practices toward COVID-19 prevention in Yemen: a community-based cross-sectional study

**DOI:** 10.3389/fpubh.2023.1178183

**Published:** 2023-07-10

**Authors:** Ola El Hajj Hassan, Ahmed Asa’ad Al-Aghbari, Maureen McGowan, Maureen Dar Iang, Huda Omer Basaleem, Khaled Abdulla Al-Sakkaf, Dalia Hyzam, Albrecht Jahn, Fekri Dureab

**Affiliations:** ^1^Heidelberg Institute of Global Health, Heidelberg University Hospital, Heidelberg, Germany; ^2^Faculty of Medicine and Health Sciences, University of Aden, Aden, Yemen; ^3^Institute of Research for International Assistance, Akkon Hochschule, Berlin, Germany

**Keywords:** attitudes, COVID-19 prevention, knowledge, practices, Yemen, emerging diseases

## Abstract

**Background:**

Pandemics, especially in fragile war-torn countries like Yemen, challenge their already strained health systems. Community adherence to pandemic prevention measures is necessary to curb the severity and spread of emerging pandemics – which is influenced by factors, such as people’s knowledge and attitudes toward the pandemic. No studies in Aden have been published on the communities’ knowledge, attitudes, and practices (KAP) toward COVID-19 prevention to date. To understand adherence to pandemic prevention measures in contexts with fragile health systems, this study investigated KAP of Yemeni participants toward the COVID-19 pandemic.

**Methods:**

We conducted face-to-face semi-structured questionnaires among 400 eligible participants whom were identified for participation in this study through systematic household sampling from eight districts in Aden, Yemen. Eligible participants were Yemeni community members who were ≥ 18 years, living for more than 10 years in Yemen, and were willing to voluntarily participate in the study. The questionnaire included questions surrounding the participants’ COVID-19 knowledge (e.g., awareness of spread and prevention), attitudes (e.g., willingness to accept the vaccine or other prevention measures), and prevention practices during the pandemic (e.g., mask wearing, social distancing, vaccine uptake). Total KAP scores were calculated. Univariate and bivariate statistical analyses were conducted using STATA 13 software.

**Results:**

From January to May 2021 we conducted 400 questionnaires with Yemeni community members. The average age was 41.5 ± 14.5 years (range 18–86 years). The results demonstrated that the participants in this study had an intermediate knowledge (53%) and fair attitude (58%) scores. However, participants reported very poor COVID-19 prevention practices- with only 11% demonstrating these practices. Only 25% (100/400) practiced social distancing, 25% (98/400) wore a mask, and only 6% (27/400) of participants accepted (at least one dose of) the COVID-19 vaccine. Factors associated with increased knowledge were being male, married, and surprisingly those having a primary and middle school education levels (*p* < 0.05). Also participants who were diagnosed with COVID-19 or had a family member diagnosed with COVID-19 (vs. those not diagnosed OR = 2.08, 95% CI 1.07–3.78, *p* < 0.05) were more likely to know that the vaccine protects against severe COVID-19 infection and were more likely to apply good practices such as accepting the vaccine (OR = 2.65, 95% CI 1.17–6.00, *p* < 0.05) compared to those who were not.

**Conclusion:**

These findings raise awareness for the need of community-oriented education programs for COVID-19 which considers associated factors to improve the level of public knowledge, attitudes, and practices.

## 1. Introduction

Coronavirus disease (COVID-19) is a threat to global health and was declared a public health emergency of international concern as well as a global pandemic in March 2020 (1). COVID-19 is a respiratory disease that is highly transmissible and has been recorded among 627 million people globally and has attributed to more than 6,578,440 deaths as of November 1st, 2022 (2). COVID-19 is caused by infection with a novel and highly contagious coronavirus strain (SARS-CoV-2) that spreads predominately though respiratory droplets. It was first identified in Wuhan, China in 2019 (3, 4). The frequent modes of COVID-19 transmission include (1) contact with droplets produced when an infected person coughs or sneezes and/or (2) when in contact with contaminated surfaces (5). While many infections are asymptomatic, COVID-19 can progress to severe illness such as pneumonia, acute respiratory distress syndrome (ARDS), multi-organ dysfunction, and ultimately death (6). People who are of older age and those with co-morbid chronic disease are at higher risk of serious and critical illness (1, 7).

Strategies to control the spread of COVID-19 include following certain preventative measures such as early screening, early diagnosis, isolation, and treatment of cases (8). Moreover, additional strategies to control infections at community level are through frequent hand washing, hand sanitizing, and social distancing (8). To guide these prevention strategies, the World Health Organization (WHO) adopted a one year Strategic Preparedness and Response Plan (SPRP) which intended to help direct the public health response to the pandemic at different phases and to prioritize the worldwide strategic response to COVID-19 (9).

Pandemic diseases pose a challenge to health systems, especially in war-torn countries like Yemen which was already strained by more than seven years of civil conflict, mass human displacement, natural disasters, and several disease outbreaks (e.g., cholera, diphtheria, and measles) (10–12). In Yemen, the first confirmed case of COVID-19 was on April 10^th^, 2020 in Hadhramaut (12). Recent data shows that Yemen has since then registered 11,939 confirmed cases and 2,158 deaths attributed to COVID-19 (13). However, the real burden and trajectory of the disease and its spread are vague, due to the absence of well-equipped labs, testing tools, human capacity, and poor infrastructure challenging effective monitoring and evaluation of the COVID-19 pandemic (14). Similarly, limited human capacity and medical supplies may have attributed to inconsistencies in the numbers of confirmed COVID-19 cases and deaths reported. For example, one geospatial analysis of burial activity in Aden during the pandemic, suggested the extensive (and under reported) impact of COVID-19 mortalities (15). The findings in the study estimated ~1,500 excess burials across Aden by July 6th, 2020 and up to 2,120 excess burials by September 19th, 2021. At the peak of the pandemic, they found an increase in burials of nearly 230% compared to the previously reported numbers (15).

For effective control and mitigation of further COVID-19 infections, adherence to protective and preventive measures are necessary. Specifically, the compliance with protective health measures is often influenced by the population’s knowledge, attitude, and practice (KAP) (16, 17). For example, studies conducted during the severe acute respiratory syndrome (SARS) outbreak in 2003 in China and the US found that population attitudes and practices toward the SARS virus prevention were influenced by negatively held emotions and assumptions (18–20). Moreover, COVID-19 knowledge gaps have been associated with multiple socio-economic patterns (e.g, limited education, and low income)- and individuals who demonstrated both were found to undermined the risk of COVID-19 spread and symptoms (20).

This study is the first to identify the knowledge gaps and practices toward COVID-19 prevention among a community in Yemen. We aim to generate actionable and timely data from this population to inform tools that are needed to gauge the public’s awareness of and attitudes toward the disease. This study will also help inform health authorities to craft robust interventions and effective policies regarding the management of COVID-19 that are relevant and appropriate to the Yemeni situation.

## 2. Materials and methods

### 2.1. Setting

**Figure 1:**
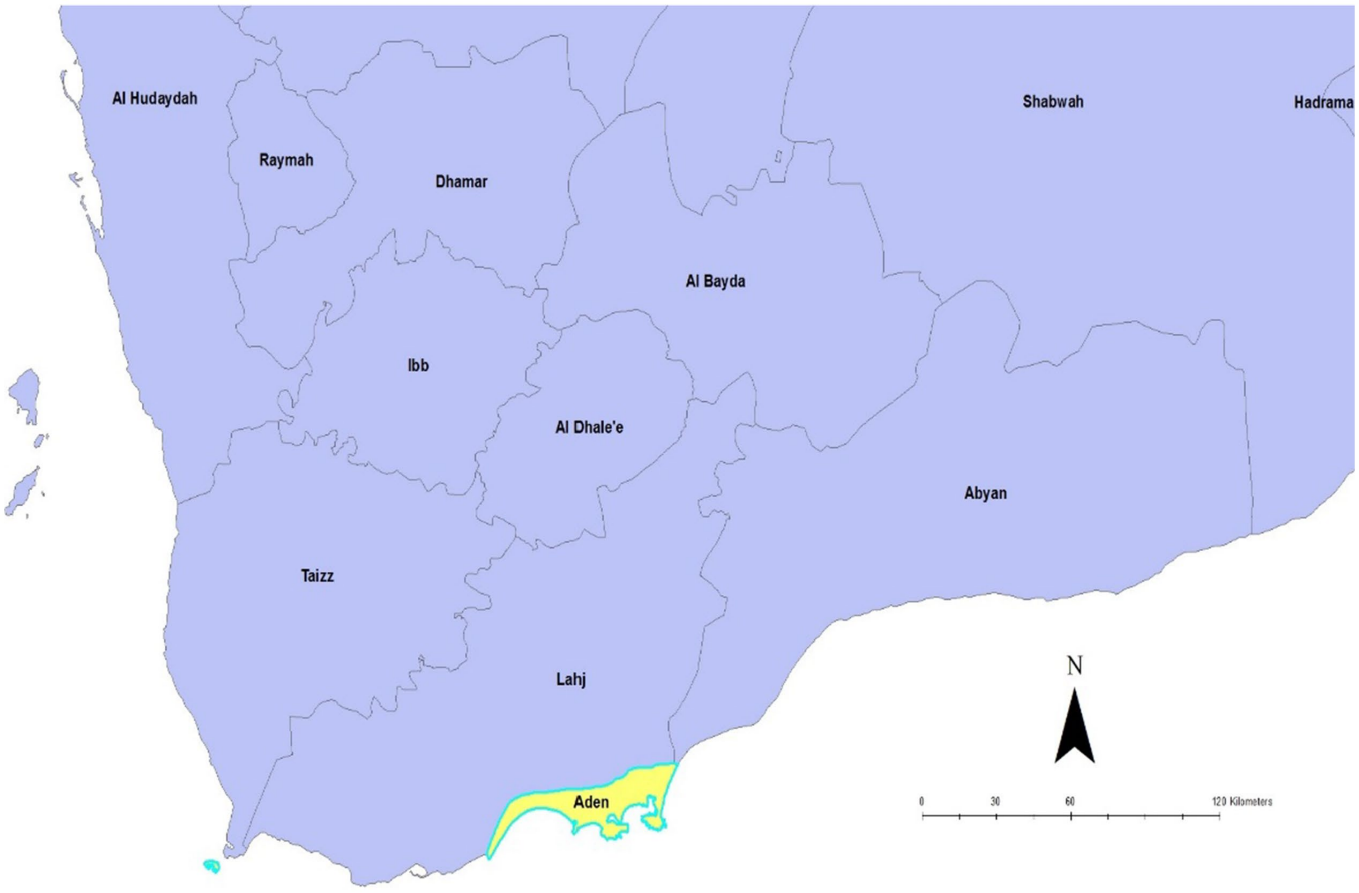
Source: Dureab F et al. Map showing location of Aden, Yemen. Aden; 2019 (21).

Yemen has 22 governorates, this study was conducted in the Aden governorate which has eight districts: Sira, Khormaksar, Al Mualla, Al Tawahi, Sheikh Othman, Mansoura, Dar Saad, and Brega. Each district has its own focal health point, which reports to the central Ministry of Public Health and Population (MoPHP) through the Governorate Health Office (GHO). In this study, the Aden governorate was selected for the following reasons: Aden has a large catchment area covering 1,114 km^2^, a population of around one million people, and it was the first governorate in Yemen to register COVID-19 cases (Figure 1).

### 2.2. Study design and sampling

A cross-sectional study was conducted between January and May 2021. Four of the eight districts were selected by simple random sampling. Participants were randomly selected based on cluster sampling. The sample size (N) needed for the study was calculated using the formula *N* = Z2P (1–P)/d2 with the assumption that the proportion of Yemeni women with health (maternal and neonatal) knowledge (P) was 50% with a 5% margin of error (d) and a 95% confidence interval (CI; Z) equal to 1.96 as based on previous literature (22).

A simple systematic sampling method was used to recruit participants- 100 from each district (total of 400 participants). Of households selected to participate in this study, the first and third household respondents were randomly selected. The respondents who met the inclusion criteria and agreed to participate in the study were recruited and this process was continued until the calculated sample size was reached.

### 2.3. Data collection tool

The data was collected by eight trained research assistants using a face-to-face semi-structured questionnaire in the local language (Arabic). The questionnaire was adapted from a similar KAP study on COVID-19 conducted by Zhong et al. (2020) in China. We piloted the questionnaire among experts at the University of Aden and Heidelberg University before being implemented. A three-day training course was provided to eight research assistants by Aden’s research team to develop their interviewing skills and ensure consistency in the data collection. The questionnaire collected information on (1) socio-demographic characteristics; (2) knowledge of COVID-19 transmission and prevention measures; (3) attitudes toward COVID-19 prevention measures; and (4) practices regarding COVID-19 prevention. Participant’s answered most knowledge questions with “Yes/No,” or “I do not know.” Other knowledge questions were open-ended which included asking participants to list forms of COVID-19 transmission and prevention methods they utilized. For both types of questions (closed and open-ended) those who gave an incorrect or an incomplete answer was scored “0,” and the correct answer was scored “1.” Similarly, in the section on attitudes and practices, a score of “1” was given to answers that reflected positive attitudes and/or good prevention practices toward COVID-19 and a score of “0” was given to answers that reflected negative attitudes and/or poor COVID-19 prevention practices. Practices were deemed good/poor through comparison with guidelines and literature on COVID-19 prevention practices. Two researchers, both with graduate-level quantitative training, agreed on categorizing answers as good/poor via consensus. Thereafter, an average score was calculated for each section and compared with a predetermined scale: 0–40% = poor knowledge/negative attitudes/ poor practices; 41–70% = intermediate knowledge/fair attitudes/fair practices and 71–100% = good knowledge/positive attitudes/good practices.

### 2.4. Validity and reliability of the data collected

The validity of this study was assured by the following actions. The semi-structured questionnaire demonstrated both content validity and face validity through its adaptation from a recent study (23). Furthermore, face validity was established by having four researchers – two from each university (with clinical and/or academic backgrounds) review the questionnaire and confirm that it measured the variables of interest. Thereafter, the questionnaire was reviewed by a German international health expert (study supervisor), three additional Yemeni researchers, and two medical doctors before implementation. To assure efficacy of the questionnaire, it was piloted with 20 Yemeni community members selected with the same age criteria (≥18 years) and who maintained similar socio-demographic characteristics to the participants in the final study. Based on this pilot study, two questions were modified.

### 2.5. Statistical analysis

All data was translated from Arabic to English by an experienced translator, cleaned, and coded using Excel. Descriptive analyses, standard deviations for continuous variables, and the count/percentages for the dichotomous or categorical variables were analyzed in Stata^®^ 13 software. Descriptive statistics were presented in four main sections: (1) socio-demographic characteristics; (2) knowledge of COVID-19 transmission and prevention methods; (3) attitudes toward adherence to COVID-19 prevention measures; and (4) practices in regard to COVID-19 prevention and vaccination uptake.

Inductive statistics using chi-squared tests, *p*-values, and linear logistic models (odds ratios) were conducted to analyze the association between all socio-demographic variables (age, education, marital status, and occupation, time to reach health facility) and variables reflecting participant knowledge, attitudes and practices toward COVID-19 prevention was presented using tables. Analysis of variance and chi-squared tests were used to measure significance. The level of significance was set at *p* < 0.05.

## 3. Results

A total of 400 participants completed the questionnaire (response rate = 100%) and participated in this study with a mean age of 41 (SD 15) years and a range of 18 to 86 years. More than half the participants were males (*n* = 205, 51%) and most participants were married (*n* = 267, 67%). The majority of participants (*n* = 265, 66%) had either completed a high school education or had a university degree. Fewer participants had either completed middle school (*n* = 42, 10%), elementary school (*n* = 47, 12%), or had had no formal education (*n* = 37, 9%). About half of the participants were employed (*n* = 203, 50.75%) and only about a quarter of participants (*n* = 106, 26%) reported that they or one of their family members were diagnosed with COVID-19. Further, the majority of participants (*n* = 267, 66%) said that they need less than 15 min to reach the closest health facility. Table 1 presents the detailed socio-demographic characteristics of the participants.

**Table 1:** Respondents socio-demographic and health characteristics in Aden (*N* = 400).

Characteristics	Frequency (*N*)	Percentage (%)
*Age*		
≤24	54	13.5
25–49	218	54.5
≥50	128	32.0
*Gender*		
Female	205	51.3
Male	195	48.7
*Marital Status*		
Married	267	66.8
Other	133	33.3
*Occupation*		
Employed	203	50.7
Unemployed	197	49.3
*Education*		
Elementary school	46	11.5
Up to middle School	89	22.3
High School/University	265	66.3
*Time to reach health facility*		
Less than 15 min	267	66.8
15 to 30 min	115	28.8
More than 30 min	18	4.50
*Has been diagnosed with COVID-19 (participant or one close family member)*		
No	286	71.5
Yes	106	26.5
I do not know	8	2.00

### 3.1. Participant knowledge on COVID-19

Table 2 shows results of participants’ knowledge on COVID-19 cause of infection, onset, transmission modes, and prevention methods. The majority of participants (*n* = 286, 72%) in this study reported that they received information on COVID-19, mainly from media/news (*n* = 288, 72%) and family/friends (*n* = 195, 49%). The majority of participants (*n* = 320, 80%) correctly reported that COVID-19 is a disease caused by a virus. Around 64% (*n* = 255) of participants correctly answered that symptoms appear between 2–14 days and 79% (*n* = 314) of participants understood the disease can be fatal. Moreover, the majority of participants (*n* = 345, 86%) reported that they knew how COVID-19 is transmitted, however, only the minority of respondents identified that contact with other individuals (*n* = 81, 20%), touching surfaces (*n* = 90, 22%), coughing (*n* = 75, 18%), sneezing (*n* = 118, 29%) and droplets in the air (*n* = 77, 19%) as routes of transmission. When asked whether they knew about COVID-19 prevention methods, 91% (*n* = 364) responded “Yes.” Nevertheless, only 62% (*n* = 248) identified covering mouth and nose, 65% (*n* = 259) reported hand washing with soap and water, and 64% (*n* = 256) said social distancing were prevention methods. It is worth mentioning that only 13% (*n* = 51) of participants mentioned vaccination as methods of preventing and protecting oneself from becoming infected with COVID-19. When we asked the participants whether they think the COVID-19 vaccine is effective, 33% (*n* = 130) responded positively and the other participants either responded that the vaccination is not effective (*n* = 139, 35%) or they were unsure (*n* = 131, 33%). Participants mean score reflecting knowledge was calculated to be 53% (Table 3). Regression analysis showed that males (vs. females OR = 1.78%, 95CI 1.13–2.84, *p* < 0.05) and those ≤24 (vs. other age groups OR = 2.14%, 95CI 0.99–4.56, *p* < 0.05) were more likely to know about hand washing as a preventive measure toward COVID-19. Also, married men (vs. those having other marital statuses OR = 2.07, % 95CI 1.03–4.16, *p* < 0.05) were more likely to mention vaccination as a preventive measure. Surprisingly, those participants having lower education level (primary school and middle school) were more likely to have adequate knowledge on preventive measures such as covering their mouth and nose (OR = 3.06, 95% CI 1.52–6.13, *p* < 0.05,OR = 3.23, 95% CI 1.92–5.43, *p* < 0.01), hand washing (OR = 6.85, 95% CI 3.3–14.2, *p* < 0.001, OR = 1.70, 95% CI 1.01–2.89, *p* < 0.05), social distancing (OR = 5.46, 95% CI 2.66–11.24, *p* < 0.01, OR = 2.03, 95% CI 1.21–3.42, *p* < 0.01), and vaccination (OR = 4.01, 95% CI 1.09–14.70, *p* < 0.05, OR = 18.59, 95% CI 2.49–138.5, *p* < 0.01) compared to those with higher education level (high school and university degree participants). Additionally, those needing less than 15 min to reach the closest health facilities (vs. those that need more than 15 min OR = 0.43, 95% CI 0.27–0.68, *p* < 0.01, OR = 0.62, 95% CI 0.39–0.98, *p* < 0.05, OR = 0.56, 95% CI 0.35–0.88, *p* < 0.01) were less likely to mention covering mouth and nose, hand washing and social distancing as methods of prevention toward COVID-19. Finally, those who were diagnosed with having COVID-19 or had a family member diagnosed with COVID-19 (vs. those not diagnosed OR = 2.08, 95% CI 1.07–3.78, *p* < 0.05) were more likely to know that the vaccine protects against severe COVID-19 infection (Table 4).

**Table 2:** Knowledge of COVID-19, transmission, and prevention (*N* = 400).

Knowledge items	Correct response *N* (%)	Incorrect response *N* (%)
Ever received information on COVID-19*	286 (71.50)	106 (26.50)
Causative Agent of Covid-19	320 (80.00)	80 (20.00)
COVID-19 symptoms appear in 2–14 days	255 (63.75)	145 (36.25)
COVID-19 can be fatal	314 (78.50)	62 (15.50)
Know tramission routes of COVID-19	345 (86)	55 (14)
Contact with others	81 (20.25)	319 (79.75)
Touching surfaces	90 (22.50)	310 (77.50)
Coughing	75 (18.75)	325 (81.25)
Sneezing	118 (29.50)	282 (70.50)
Through the air	77 (19.25)	323 (80.75)
Know main prevention methods	364 (91)	36 (9)
Cover mouth and nose	248 (62.00)	152 (38.00)
Hand washing with water and soap	259 (64.75)	141 (35.25)
Social Distancing	256 (64.00)	144 (36.00)
Vaccination	51 (12.75)	349 (87.25)
Believe protective measures are effective*	293 (73.25)	59 (14.75)
Believe COVID-19 vaccine is protective	130 (32.50)	270 (67.50)

* Numbers for each item may not add up to the total number of study population due to missing values.

**Table 3:** Mean score of grouped variables reflecting knowledge, attitudes, and practices.

Sections	Correct/Positive answers	Number of total participant answers	Score (%)	Interpretation
Knowledge	3,562	6,720	53	Intermediate
Attitudes	700	1,200	58	Fair
Practices	581	5,200	11	Poor

**Table 4:** Attitudes toward COVID-19 prevention and vaccination (*N* = 400).

Attitude items	Correct response *N* (%)	Incorrect response *N* (%)
Needs more information on COVID-19	177 (44.25)	223 (55.75)
Would take the vaccine	157 (39.25)	243 (60.75)
Would commit to prevention methods	366 (91.50)	34 (8.50)

### 3.2. Participant’s attitudes toward COVID-19 prevention and vaccination

Only 44% (*n* = 177) of the participants in this study reported interest in receiving more information on COVID-19. However, the majority of participants (*n* = 366, 92%) showed that they were willing to comply with the COVID-19 restrictions such as lockdowns, if established. Interestingly, around 61% (*n* = 243) of the respondents were not willing to accept the COVID-19 mainly for the following reasons: there were many rumors about the vaccine (*n* = 55.13%), the vaccine was believed to cause other health problems (*n* = 53, 13%); participants were unwilling to try a novel vaccine (*n* = 48, 12%); the vaccine was considered to be a conspiracy (*n* = 32, 8%); or the belief that the vaccine would not prevent infection (*n* = 28, 7%). Participants’ mean attitude score was 58% (Table 3). Regression analysis showed that males (vs. females OR = 1.62, 95% CI 1.03–2.54, *p* < 0.05), participants between 24–49 years of age (vs. other age groups OR = 2.32, 95% CI 1.39–3.89, *p* < 0.001) were more likely accept to take the vaccine if it was offered to them. However, those with low education levels such as elementary school OR = 0.31, 95% CI 0.13–0.74, *p* < 0.01) and those with middle school education (vs. High education OR = 0.51, 95% CI 0.29–0.89, *p* < 0.05) were less likely to accept to take the vaccine if offered to them (Table 5).

**Table 5:** Results of logistic regression analysis on factors associated with adequate knowledge, positive attitudes, and good practices toward COVID-19.

Variables	Good knowledge			Positive attitudes		Good practice
	Cover Mouth and Nose	Hand washing	Social Distancing	Vaccination	Would you take the Vaccine?	Did you take the vaccine?
	OR (95% CI)	OR (95% CI)	OR (95% CI)	OR (95% CI)	OR (95% CI)	OR (95% CI)
*Sex*						
Male	1.12 (0.72–1.77)	1.78 (1.13–2.84)*	1.32 (0.83−2.09)	1.48 (0.78–2.79)	1.62 (1.03–2.54)*	1.11 (0.49–2.54)
Female	Ref	Ref	Ref	Ref	Ref	Ref
*Age*						
<24	1.10 (0.51–2.38)	2.14 (0.99–4.56)*	1.22 (0.57−2.62)	2.66 (0.87−8.14)	1.29 (0.58–2.87)	2.46 (0.54–11.19)
24–49	1.03 (0.63–1.67)	0.88 (0.64–1.44)	0.96 (0.59–1.57)	1.40 (0.69–2.85)	2.32 (1.39–3.89)***	1.60 (0.66–3.86)
>50	Ref	Ref	Ref	Ref	Ref	Ref
*Marital status*						
Married	0.76 (0.46–1.25)	0.931 (0.56–1.56)	0.66 (0.39–1.08)	2.07 (1.03–4.16)*	0.75 (0.45–1.26)	1.88 (0.78–4.49)
Others		Ref	Ref	Ref	Ref	Ref
*Occupation*						
Employed	0.79 (0.51–1.24)	0.99 (0.63–1.56)	0.82 (0.53–1.30)	0.71 (0.37–1.37)	1.16 (0.74–1.83)	1.34 (0.57–3.11)
Unemployed	Ref	Ref	Ref	Ref	Ref	Ref
*Educational level*						
Elementary School	3.06 (1.52–6.13)*	6.85 (3.3–14.2)***	5.46 (2.66–11.24)***	4.01 (1.09–14.70)*	0.31 (0.13–0.74)**	0.89 (0.26–3.08)
Middle school	3.23 (1.92–5.43)**	1.70 (1.01–2.89)*	2.03 (1.21–3.42)**	18.59 (2.49–138.55)**	0.51 (0.29–0.89)*	2.17 (0.61–7.7)
High education level	Ref	Ref	Ref	Ref	Ref	Ref
*Health facility access*						
<15 min	0.43 (0.27–0.68)**	0.62 (0.39–0.98)*	0.56 (0.35–0.88)**	1.49 (0.78–2.88)	0.65 (0.41–1.04)	0.45 (0.17–1.21)
>15 min	Ref	Ref	Ref	Ref	Ref	Ref
*Diagnosed with covid-19*						
Yes	0.99 (0.61–1.61)	1.10 (0.67–1.79)	1.48 (0.89–2.44)	2.08 (1.07–3.78)*	1.08 (0.67–1.74)	2.65 (1.17–6.00)*
No	Ref	Ref	Ref	Ref	Ref	Ref

OR, odds ratio. CI, confidence interval. *p < 0.05, **p < 0.01, ***p < 0.001.

### 3.3. Participant’s practices taken for the prevention of infection

Table 6 describes the protection measures taken by participants to prevent COVID-19 infection. A quarter of the participants applied social distancing (*n* = 100, 25%); covered their mouth and nose (*n* = 98, 24.5%); (*n* = 93, 23%); followed hand washing etiquette; used sterilization (*n* = 76, 19%); or avoided close contact with others (*n* = 34, 9%), as methods to prevent infection. Some participants mentioned additional methods they employed to prevent infection such as eating healthy foods; drinking herbal teas and juices; inhaling steam, and taking vitamins. Interestingly, only around 1% (*n* = 5) of participants said that they were fully vaccinated against COVID-19. However, when asked in a separate question, only 6% (*n* = 27) of the participants took at least one dose of the COVID-19 vaccine. Participants’ mean practice score was 19% (Table 3). Regression analysis showed that those who were personally diagnosed or had a family member diagnosed with COVID-19 (vs. those who were not diagnosed OR = 2.65, 95% CI 1.17–6.00, *p* < 0.05) were more likely to have accepted the COVID-19 vaccine (Table 5).

**Table 6:** Preventive practices taken by respondents during the COVID-19 pandemic (*N* = 400).

Practice items	Correct response *N* (%)	Incorrect response *N* (%)
Practice social distancing	100 (25.00)	300 (75.00)
Wear a mask	98 (24.50)	302 (75.50)
Eat healthy foods	40 (10.00)	360 (90.00)
Stay home	61 (15.25)	339 (84.75)
Avoid contact with sick people	17 (4.25)	383 (95.75)
Avoid handshaking	6 (1.50)	394 (98.50)
Avoid close contact with others	34 (8.50)	366 (91.50)
Avoid using others’ utensils	7 (1.75)	393 (98.25)
Apply hand washing	93 (23.25)	307 (76.75)
Apply hand sterilization	76 (19.00)	324 (81.00)
Take vitamins	17 (4.25)	383 (95.75)
Fully immunized	5 (1.25)	395 (98.75)
Received any dose of the vaccine	27 (6.75)	373 (93.25)

## 4. Discussion

Intermediate knowledge levels, fair attitudes, and poor practice of COVID-19 prevention methods by the public were identified in our study which may hinder proper Infection and Prevention Control (IPC) measures to mitigate COVID-19 at all levels. This is the first epidemiological study in Aden, Yemen aiming to assess the knowledge, attitudes, and practices of community members toward coronavirus and its prevention. Generating such information is crucial for promoting correct preventative actions and behaviors as a strategy to manage its spread. The findings from our study demonstrated that the study participants achieved a mean score of 53% on the correct knowledge items thereby showing intermediate levels of knowledge. The findings of this study are similar to previous studies conducted in Liberia (51.0%) (24) and Syria (60%) (25), however it is higher than that in Cameroon (21.9%) (26) but lower than that reported in some other countries such as Bangladesh (85.0%) (27) Saudi Arabia (89.9%) (28) and China (91.2%) (29). One interesting finding in our study was that the majority of participants (86%) reported that they knew about modes of COVID-19 transmission, however, only the minority gave correct answers such as contact with others (20%), touching surfaces (23%) or coughing (19%). In our sample, most participants said that they received information mainly from the media and news (67%) as opposed to social media as was found in studies conducted in Oman, Saudi Arabia, and Egypt (72, 85.8, and 80.8%). The gap in knowledge in our findings might be attributable to the fact that the population may have gained false or insufficient information about the disease and its transmission from the news, social media, and TV as daily information on COVID-19 was published during the pandemic and this study was conducted in the middle of the outbreak (14, 30, 31). Another factor that might have contributed to this difference in knowledge may be due to the difference in age distribution, as compared to other studies. For example, in Saudi Arabia more than 92% of the sample of study were under the age of 49 whereas in our study 67% of the participants were above the age of 49 (32). Our findings showed that level of knowledge on different COVID-19 topics were varied. The majority of participants in this study had information about the cause of infection, onset, and prognosis. However, the information about COVID-19 transmission and prevention was limited. Similarly, in a recent study conducted in Saudi Arabia, they found that almost half the participants did not know when and who should wear a mask to prevent infection and were unaware that COVID-19 can spread from one person to another in close proximity (30). This highlights the need for increased awareness on modes of COVID-19 transmission for preventing the spread of the virus. Surprisingly, our findings showed that those with lower education levels were more likely to know about COVID-19 preventative measures compared to those with higher education levels. This could be attributable to the fact that those with higher education levels may have critically questioned information they received from social media and other news sources (33).

Furthermore, this study highlights that less than half participants wanted to have additional COVID-19 information. Yet, the majority were willing to comply with preventive measures if established and enforced by the government such as mandatory mask wearing, social distancing, and mandatory lockdowns. Our findings are in line with attitudes from Saudi Arabia which showed that most of the community was willing to stay at home (i.e., lock down) or avoid shaking hands to mitigate the spread of the virus, if enforced by the government (32). Perhaps most importantly from our findings is that, very few respondents mentioned vaccination as a preventative and disease control measure and few participants found the vaccine effective and protective, 12.5 and 32%, respectively. Conversely, in a recent study in Oman, 52% of the participants believed that vaccines could protect them from contracting the virus and 42% believed that patients will not contract COVID-19 after vaccination (34). These differing findings may reflect the contextual factors, Oman is politically stable and is globally acknowledged for its well-structured and high coverage immunization programs while Yemen is challenged by a fragile political and healthcare system and thereby has a weak vaccination strategy (15).

In regard to vaccination, most participants (61%) in this study were not willing to accept the vaccine (61%) at the time of this study for varying reasons. The main reasons for hesitancy toward the vaccine were the presence of rumors round it (14%) and that it is a conspiracy (8%). Some participants (12%) claimed that they were not willing to try a vaccine that was newly developed and need to know its long-term effects. A systematic review conducted by Wang et al. (2021), showed that the overall acceptance rate of the COVID-19 vaccination rate was 64.1% globally ranging between 19.9 and 92.1% across countries (35, 36). Factors such as trust in the health care system and government, personal history of vaccination, concerns surrounding vaccine safety and effectiveness, concerns over rapid vaccine development, and knowledge of COVID-19 were associated with COVID-19 vaccine hesitancy (35). Our findings are in line with those from another study conducted by Nguyen et al. (2021) highlighting that *“understanding of how the vaccine works will reduce the pandemic’s consequences, especially if that understanding is shared between people.”* Confidence toward vaccine uptake may be attained when rumors are neutralized and only official sources share correct and evidence-based information (34, 37). Further, a study conducted on acceptability of COVID-19 vaccines in the Arab world, mirrored our findings in which they also found a high rate of COVID-19 vaccine hesitancy in Yemen (54%) (38). In contrast, Oman showed good level of COVID-19 vaccine acceptance in which around 59% of participants would advise others to accept it, 56.8% would take it themselves, and 47.5% were willing take a second dose (34).

When sharing their practices, only a minority of participants followed any preventative measures since the beginning of the pandemic. Only a quarter, applied social distancing, covered their mouth and nose, followed hand washing etiquette, sterilization, and/or avoided contact with others. Our findings may also reflect contextual factors in which vaccine rollout in Yemen was limited only to southern governments, and only around 2% of the Yemenis received both COVID-19 vaccine doses and around 3% received at least one dose (39, 40). Those who were personally diagnosed or had a family member diagnosed with COVID-19 (vs. those who were not diagnosed OR = 2.65, 95% CI 1.17–6.00, *p* < 0.05) were more likely to have accepted the vaccine. Yemenis’ perceived risk of accepting the vaccine largely outweighed its perceived benefits and led to limited uptake. This was due not only to personal perceptions (as found in this study) but also to external factors such limited vaccine availability and access.

This study had some limitations. The study is a cross sectional study which is a single time point study and therefore only measured KAP regarding COVID-19 at a singular time point and did not measure changing KAP over the course of the pandemic. Another limitation to this study might be through the use of dichotomous Yes/No questions which may be influenced by social desirability bias in which the participants might have provided a socially desired answer regarding their own COVID-19 prevention behaviors. While there were limitations in this study, strengths included that this is the first study conducted in Aden on COVID-19 KAP and it adds to the body of evidence on practices of COVID-19 prevention. Specifically, while current practices in Aden are poor, the community is willing to enhance and commit to prevention methods. Results of this study have significant implications for infectious disease health promotion including COVID-19 and other diseases that may spread in the future. For this reason, it is important that public health authorities set up strategies to indicate vulnerable subpopulations and prioritize policies as well as communication efforts (in collaboration with the media) to enhance public knowledge about infectious diseases. For example, the Ministry of Health can implement regular community workshops to enhance information on different emerging diseases and methods of prevention. While this is the first study to be conducted on the community in Aden regarding knowledge, attitudes, and practices surrounding COVID-19 prevention; it is important to recognize that Aden is an urban area with likely higher access to education, and thus, may not be generalizable to more rural Yemeni areas. As a result, conducting follow-up studies is recommended to understand a broader KAP toward COVID-19 in Yemen.

## 5. Conclusion

With intermediate knowledge and fair attitudes toward COVID-19 prevention, Yemen’s community has a promising chance to expand their practices on the topic of COVID-19 prevention methods. However, practice (or adoption of) these prevention methods, including vaccination rates, remains poor. This study highlights the need for increased awareness of COVID-19 modes of transmission for preventing the spread of the virus as well as the need for comprehensive and accessible vaccination campaigns. Yemen’s government and health system must establish awareness campaigns and adopt innovative communication methods to enhance the community’s knowledge of and resulting practices toward COVID-19 prevention.

## Data availability statement

The raw data supporting the conclusions of this article will be made available by the authors, without undue reservation.

## Ethics statement

The studies involving human participants were reviewed and approved by Ethical Review Committee of Heidelberg University (protocol code: S-887/2020 and date of approval: 15.02.2021) and from the Ethical Review Committee of University of Aden (protocol code: REC-96-2021 and date of approval: 23.03.2021). The patients/participants provided their written informed consent to participate in this study.

## Author contributions

OH, FD, and AJ worked on study conceptualization and methodology. FD and AA-A analyzed and interpreted data. HB, KA-S, and DH contributed to the interpretation of results and reviewed the manuscript. OH wrote the original draft. AJ supervised and contributed to writing the manuscript. AA-A, MM, and MDI reviewed and edited the manuscript. FD supervised and gave the final approval. All authors read and approved of the final manuscript.

## Funding

This project was funded by the Deutsche Forschungsgemeinschaft in Germany (DFG) (Project number: JA 967/3-1).

## Conflict of interest

The authors declare that the research was conducted in the absence of any commercial or financial relationships that could be construed as a potential conflict of interest.

## Publisher’s note

All claims expressed in this article are solely those of the authors and do not necessarily represent those of their affiliated organizations, or those of the publisher, the editors and the reviewers. Any product that may be evaluated in this article, or claim that may be made by its manufacturer, is not guaranteed or endorsed by the publisher.
